# Advances in RNA Vaccines for Preventive Indications: A Case Study of a Vaccine against Rabies

**DOI:** 10.3390/vaccines7040132

**Published:** 2019-09-27

**Authors:** Nicole Armbruster, Edith Jasny, Benjamin Petsch

**Affiliations:** 1 Scientist Prophylactic Vaccines Non-Clinical Development, CureVac AG, Paul-Ehrlich-Straße 15, D-72076 Tübingen, Germany; nicole.armbruster@curevac.com; 2 Senior Scientist Prophylactic Vaccines Non-Clinical Development, CureVac AG, Paul-Ehrlich-Straße 15, D-72076 Tübingen, Germany; edith.jasny@curevac.com; 3 Senior Director Prophylactic Vaccines Research, CureVac AG, Paul-Ehrlich-Straße 15, D-72076 Tübingen, Germany

**Keywords:** rabies vaccines, mRNA vaccines, lipid nanoparticles

## Abstract

There is a global need for effective and affordable rabies vaccines, which is unmet by current vaccines due to limitations in their production capacities, required administration schedules, storage requirements, and cost. Many different experimental approaches previously used for bacterial and viral vaccines have been applied to rabies, but with variable success. One of the most promising new concepts is the use of messenger RNA (mRNA) in encoding the main rabies virus antigen, the envelope glycoprotein (RABV-G). CureVac has applied their proprietary technology platform for the production of mRNA to this problem, resulting in the rabies vaccine candidate CV7201. Following preclinical studies in mice and pigs showing that CV7201 could induce neutralizing immune responses that protected against rabies virus, different dosages and routes of administration of CV7201 were tested in a phase 1 human study. This clinical study proved that mRNA vaccination was safe and had an acceptable reactogenicity profile, but immune responses depended on the mode of administration, and they did not unequivocally support CV7201 for further development as a prophylactic vaccine with this particular formulation. Further, preclinical studies using RABV-G mRNA encapsulated in lipid nanoparticles (LNPs) showed an improved response in both mice and nonhuman primates, and these encouraging results are currently being followed up in clinical studies in humans. This review summarizes the recent advances in mRNA vaccines against rabies.

## 1. Introduction

Rabies disease due to infection by the neurotropic rabies virus is an invariably fatal neurological disease in infected mammals, including humans. Rabies virus is a single-stranded RNA virus of the *Lyssavirus* family carried by a variety of mammalian vectors; around 95% of human infections are due to dog bites, but other vectors include coyotes, skunks, and bats [1,2]. Exposure is followed by an incubation phase and then manifests with the development of influenza-like symptoms ultimately leading to severe neurotropic symptoms caused by the ensuing progressive encephalomyelitis [3]. The duration of these phases varies, but death usually follows within 6 to 11 days after the first symptoms appear, leaving little time to attempt any therapeutic options, and the outcome is invariably death once clinical symptoms are apparent. The real burden of rabies is difficult to quantify due to under-reporting of cases and a lack of reliable surveillance data [4], but it is currently estimated that there are over 3 billion people at risk of exposure, and 59,000 people die each year due to canine rabies [5]. The unfortunate truth is that rabies kills so many people despite Louis Pasteur reporting the first successful human immunization against rabies over 130 years ago [6,7].

In that time, the vaccine development process has essentially been one of progressively improving the safety and tolerance of the inoculating substance, from a suspension of dried spinal cords from rabid rabbits in Pasteur’s first experiment to inactivated viral vaccines like Rabipur^®^ or Verorab^®^ [8]. The first widely used and effective vaccines were based on animal nerve or brain tissues, (e.g., mouse suckling brain), which caused severe side effects due to hypersensitivity to the myelin contaminant [9]. Egg-based culture systems used to grow viruses brought their own problems of egg allergies, and further work resulted in viral propagation in chick embryos, MRC-5, human diploids, and, most recently, Vero cell-culture systems. These approaches have all resulted in efficacious vaccines, typically administered in a three-dose schedule for pre-exposure prophylaxis, but at the cost of slow, tedious, and expensive production in limited quantities. Moreover, manufacturing requires handling of infectious rabies virus with respective specific precautions. Currently, these licensed vaccines are widely used by those at risk in Europe and North America, for example, animal handlers, veterinarians, forestry workers, and for travelers to high-risk regions, although the latter display poor compliance with the three injection primary vaccination series [10].

However, rabies is most prevalent in low- and middle-income countries where the virus is endemic in dogs [5]. The high cost of current rabies vaccines limits their accessibility, and, consequently, vaccination coverage rates and full compliance with recommended schedules in such countries is low, particularly among children who suffer a high burden of disease. Cost and supply constraints have led some Latin American and Asian countries to continue using nerve tissue vaccines against WHO recommendations [3,11]. Postexposure prophylaxis (PEP) for suspected rabies exposure also consists of rabies vaccination combined with administration of anti-rabies immunoglobulins (RIG) [12]. Although effective, there have been cases of PEP failure in developing countries where the disease burden is highest due to unaffordable treatment costs [13]. This has led to investigations of intradermal administration of fractional dose schedules to reduce the cost [14].

### 1.1. Innovative Approaches with Recombinant Technology

There is clearly an urgent need for more affordable, effective, and safe rabies vaccines utilizing new technologies. The rabies virus genome is relatively simple, encoding five structural proteins—glycoprotein (G), nucleoprotein (N), phosphoprotein (P), matrix protein (M), and RNA polymerase (L) [15]. Of these, the envelope glycoprotein acts as an antigen to induce virus-neutralizing antibodies, which have been shown to be protective in animal challenge models [16]. Recombinant technology has been applied to the expression of glycoprotein in a variety of cell systems including mammalian cells (Vero cells, human embryonic kidney cells, baby hamster kidney cells, and Chinese hamster ovary cells), insect cells, yeasts, and plants [17]. All these systems require extensive processing of the expressed protein, with purification and additional postprocessing steps, which, together with additional requirements for adjuvantation, may make them economically nonviable. An alternative to using recombinant technology to produce the protein antigen is the use of characterized glycoprotein mRNA or DNA as the immunogen itself. DNA plasmids and messenger RNA (mRNA) can be administered and taken up by cells where they are used to translate the encoded target antigens and induce an immune response.

### 1.2. DNA Vaccines

The principle of DNA vaccines has been confirmed for rabies virus in small animal models in which they demonstrate immunogenicity [18], but this has not translated into effective vaccines in larger animals, and they are poorly immunogenic in humans [19]. Further, there is concern, so far with no supporting evidence, that exogenous DNA could be incorporated into the host genome.

### 1.3. mRNA Vaccines

An alternative is the use of mRNA technology to easily produce mRNA that is highly specific for the encoded antigen and presents several advantages over existing vaccine technologies for the development and production of prophylactic vaccines against infectious diseases. Since, unlike live or attenuated vaccines, mRNA vaccines do not introduce any living virus material into the vaccinees, they do not pose the safety risk of reversion to pathogenicity and, hence, infection [20]. In addition, they do not interact with the host-cell DNA, so they avoid the potential risk of genomic integration posed by DNA-based vaccines, but can still encode any protein antigen of choice, offering a large potential for the development of prophylactic vaccines against known and as yet unidentified pathogenic threats. As mRNA binds to pattern recognition receptors, mRNA vaccines induce the innate immune system, a property which peptide- and protein-based vaccines lack if not supplemented with an adjuvant. The degree of innate stimulation by mRNA vaccines is dependent on the chemical nature of the nucleotides and strongly influenced by the formulation [21,22]. Furthermore, mRNA vaccines can be administered by traditional intradermal or intramuscular routes using conventional needle-based injections and, unlike DNA vaccines, they do not require any additional administration device such as electroporation. As many different proteins can be encoded, mRNA vaccines also offer the maximum flexibility with respect to development and manufacturing processes [23]. Since differences in the encoded protein only require alteration of the sequence of the mRNA molecule, leaving its physicochemical characteristics largely unaffected, the mRNA vaccine platform can use the same purification techniques and equipment to manufacture vaccines protecting against different pathogens, saving both time and costs compared with other vaccine platforms. As mRNA production does not require a dedicated product-specific facility, this obviates the need for the strict containment measures that are applied to other vaccines that require viral growth. For example, most of the current global poliovirus disease burden is due to vaccine-derived polioviruses (VDPVs), the live attenuated viruses in oral polio vaccines reacquiring neurovirulence [24]. The greatest risk of reappearance of poliovirus following eradication will be from vaccine manufacturing facilities handling large quantities of infectious polioviruses, necessitating rigorous containment conditions [25]. In contrast, mRNA vaccines offer a flexible one-for-all, large-scale, safe, and cost-effective manufacturing process with a fast turnaround time.

## 2. Application of mRNA Technology to Rabies

There is extensive ongoing research by multiple scientists and companies into the use of mRNA as prophylactic vaccines for infectious diseases. We have been applying our expertise with mRNA to target different infectious diseases employing a range of additional technologies to augment and direct the immune response [26,27]. For this purpose, we employ an mRNA technology that uses sequence-optimized, unmodified mRNA as the basis for vaccine development. Using in situ protein expression, mRNA vaccines are capable of inducing a balanced immune response comprising both cellular and humoral immunity while not being subject to major histocompatibility (MHC) haplotype restriction. There is a well-established Good Manufacturing Practice process for manufacturing clinical grade material through preparation of functional synthetic mRNA by in vitro transcription of a cDNA template, typically plasmid DNA (pDNA). The linearized pDNA template is transcribed into mRNA in a mixture containing recombinant T7 RNA polymerase and nucleoside triphosphates. Following transcription, the pDNA template is digested by DNase and purified by high performance liquid chromatopraphy to remove impurities. The final synthetic mRNA contains a protein-encoding open reading frame (ORF) flanked by two elements essential for the function of mature eukaryotic mRNA: a 5′ Cap and a poly(A) tail at the 3′ end, as well as 5′ and 3′ untranslated regions (UTRs), which increase translation and stability [28]. The optimization of the ORF entails GC enrichment using a proprietary algorithm.

Rabies was chosen as our first target as a prophylactic mRNA vaccine to move through preclinical animal models before progressing to assessments in humans. The decision to initially target rabies was dictated by several factors. As previously noted, the rabies genome is simple and well characterized as is the target of the immune response, the RABV-G protein antigen [29]. As there are several licensed comparator vaccines available, and validated standardized tests for measurement of the immune response to the RABV-G antigen exist, rabies mRNA vaccine candidates can be readily compared with licensed and commercially available rabies vaccines [30]. Further, a consequence of the inevitably fatal outcome of natural infection with rabies virus means that any human volunteers will be naïve to the virus unless they have previously been vaccinated, whereas most humans have some history of exposure to other potential viral pathogen targets like seasonal influenza viruses. Another reason why rabies is a good target to model a vaccine in development is the availability of a WHO-accepted threshold of protection based on the functional immune response, which can already be evaluated in phase 1 trials of a clinical development program [11]. These factors all combine to allow relatively small clinical trials of the vaccine, thus simplifying and accelerating clinical development. Rabies will also serve as a model antigen to fully investigate and understand the mRNA platform, which will help to establish a database of its safety and tolerability as well as to predict immunogenicity potential for other mRNA vaccines, with the ultimate goal being the ability to accelerate development of other vaccines for which there is a recognized unmet medical need.

### 2.1. mRNA Vaccine Candidate CV7201: Preclinical Studies

The first mRNA rabies vaccine candidate, CV7201, is a lyophilized, temperature-stable mRNA composed of free and complexed mRNA encoding the rabies virus glycoprotein (RABV-G) formulated with the cationic protein protamine as stabilizer and adjuvant. The feasibility of an mRNA rabies vaccine based on nonchemically modified nucleotide technology was originally demonstrated in a series of experiments in mice and then in adult and newborn pigs by Schnee et al. [31]. In BALB/c mice, two doses of RABV-G mRNA administered by intradermal injection, 21 days apart, produced rabies-virus neutralizing antibody titers (VNTs) considered protective in cats, dogs, and humans (≥0.5 IU/mL) [11]. The responses in in Balb/c mice were durable, with antibodies persisting for a year after mRNA administration, and dose-dependent from 1.25 to 80 μg mRNA; doses of 10 μg and higher induced VNTs ≥0.5 IU/mL. Responses of similar magnitude were obtained in mice following intramuscular injection of fractional doses of human rabies vaccines—human diploid cell rabies vaccine (HDC) or the purified chick embryo cell culture vaccine (PCEC) Rabipur [30,31].

BALB/c mice typically produce Th2 helper cells, which will ensure an antibody response, but similar results were obtained in C57BL/6 mice in which Th1 responses predominate and, thus, typically have better cellular immune (CMI) responses [32]. Comparing CMI responses to RABV-G mRNA and Rabipur revealed similar induction of rabies-specific CD8+ cells but a higher CD4+ T cell response with the mRNA than the licensed vaccine. In depletion experiments, these CD4+ T cells were found to be necessary for the induction of virus-neutralizing antibodies. These CMI responses to mRNA were also dose-dependent and of long duration [31].

**Figure 1:**
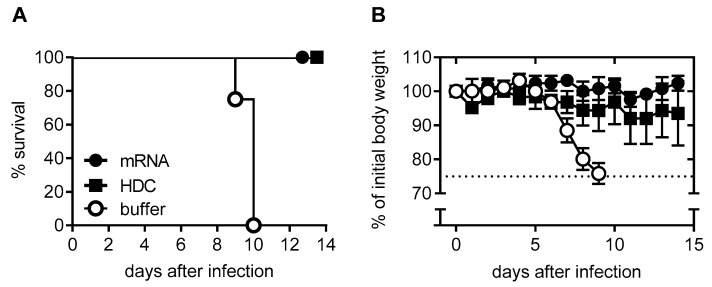
Protective capacity of mRNA vaccine against lethal intracerebral (i.c.) rabies challenge infection in mice (adapted from reference [31]). (**A**) shows the survival curve of three groups of female BALB/c mice (*n* = 5 per group) who received two doses of 80 μg RABV-G mRNA, 0.1 human dose of human diploid cell rabies vaccine (HDC), or injection buffer 21 days apart. Six weeks later, they were infected i.c. with rabies virus. (**B**) shows the mean body weight (±SD) of challenged animals. Animals were euthanized if they lost 25% of body weight.

In the domestic pig, which shares many physiological characteristics with humans, two intradermal doses of RABV-G mRNA elicited 100% seroconversion [31]. All animals had neutralizing titers above the WHO threshold (>0.5 IU/mL), with no further increase after a third vaccination. As in mice, these responses were dose-dependent and similar in magnitude to those elicited by Rabipur. Schnee et al. [31] also described challenge studies in BALB/c mice. Intracerebral injection of rabies virus strain CVS-11 at a dose 40-fold greater than the median lethal dose (LD_50_) resulted in weight loss and death in all untreated animals and was used as a potency test for rabies vaccine release [33]. Animals were pretreated with two 80 μg doses of mRNA or one-tenth the human dose of HDC vaccine and then challenged six weeks after the second dose. Untreated controls started to show weight loss five days after challenge, and all had died or were euthanized by day 10 (Figure 1). Two animals pretreated with HDC displayed 10–20% weight loss, but they survived, while all mRNA-treated animals survived without any weight loss. Examination of the brains of challenged animals for evidence of rabies viral replication at the telencephalon revealed that this was suppressed in both mRNA- and Rabipur-treated animals [31].

### 2.2. CV7201 in Humans

The encouraging data from the preclinical animal studies led Alberer et al. [34] to conduct the first in-human demonstration of the safety and immunogenicity of CV7201 (ClinicalTrials.gov: NCT NCT02241135). The vaccine CV7201 displayed an acceptable safety profile, but immune responses depended on mode of administration. When using a needle-free injection device, we were able to induce titers close to or above the WHO threshold of 0.5 IU/mL [34]. In this phase 1 study, 101 male and female adults received 80 to 640 μg doses of CV7201 on days 0, 7, and 28 or days 0, 28, and 56 by intramuscular or intradermal injection using three different needle-free injection devices or conventional needle-syringe based injection.

The primary study objective was the safety and reactogenicity of the injected mRNA assessed by soliciting local (injection site) reactions and systemic adverse events (AEs) for seven days after administration of each dose and noting any serious adverse events (SAEs). In this small study, the vaccine appeared to be safe; of three SAEs reported, only one—a case of transient moderate Bell’s Palsy—was considered to be possibly related to vaccination. Some degree of transient injection site pain was reported by 94% of intradermally injected subjects as well as 97% of intramuscularly injected subjects, but these events were all considered to be mild or moderate by the vaccinees. Other local reactions were more frequent with intradermal than intramuscular injections, and they were also mainly mild and transient. Systemic AEs were less frequent, occurring in 78% of vaccinated subjects, while 5% were described as severe. There was no clear association with any particular dose or route of administration, and all resolved without sequelae, so reactogenicity was considered acceptable. Unsolicited AEs reported up to four months after vaccination were typical disorders observed in vaccine trials with only nasopharyngitis, headache, oropharyngeal pain, vertigo, and rhinitis being reported by 5% or more of the participants.

Humoral responses to mRNA were measured using a standardized WHO recommended rapid fluorescent focus inhibition test (RFFIT) for rabies antibodies [35]. Immunogenicity across the different groups was clearly affected by the route of administration. Of 18 subjects given CV7201 intradermally using a needle syringe, only one subject, who received three injections of the highest dose (320 μg), had detectable antibodies. Similarly, no subjects given 80 to 640 μg by intramuscular injection using needle syringes displayed any response, whereas 10 of 13 subjects (77%) who received three 200 or 400 μg doses by intramuscular injection using an injector (Pharmajet Stratis™) displayed antibodies, six of these achieving the WHO predefined titer (≥0.5 IU/mL).

**Figure 2:**
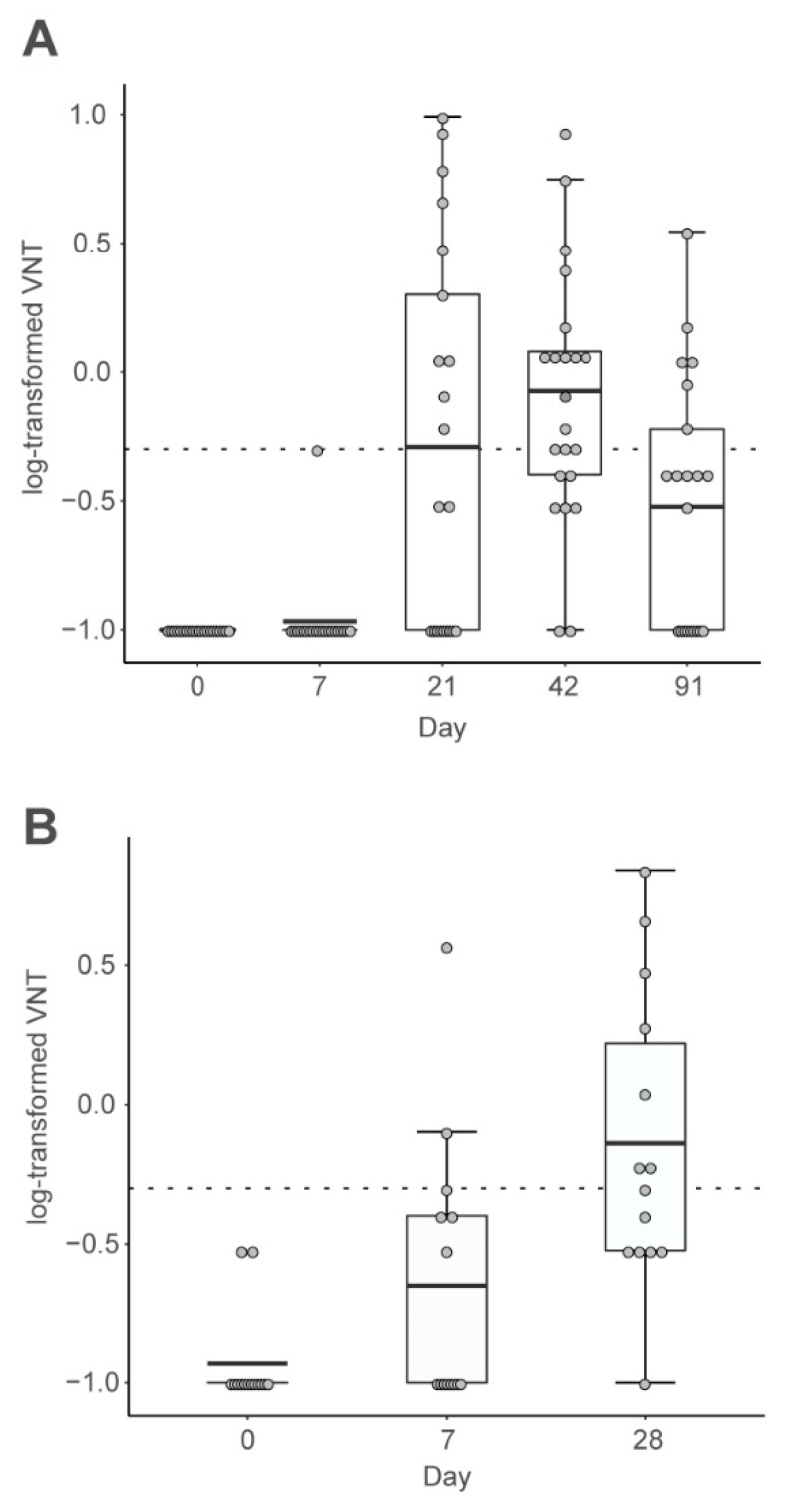
Serum neutralizing antibody responses in human volunteers to intradermal injection of 80 μg mRNA using a Tropis needleless injector (adapted from reference [34]). (**A**), responses to three primary vaccinations (*n* = 21), (**B**), responses to the one-year booster (*n* = 14). Each dot represents a participant. Boxes depict geometric mean titers and interquartile ranges (IQRs); whiskers extend from the hinge to the highest or lowest value within 1.5 × IQR of the respective hinge, and the dashed line marks the protective threshold. VNT = virus neutralizing titer.

In contrast, most subjects who received three 80 or 160 μg doses by intradermal injection using a needleless injector device (Pharmajet Tropis ID™) displayed increased antibodies after the second dose (Figure 2A). In most cases (25/33, 76%) titers achieved were above the WHO predefined level (≥0.5 IU/mL). One year later, antibodies were no longer detectable, but 6 of the 14 subjects (43%) displayed a response within 7 days of a single booster dose, and by day 28 postboost, 13 of the 14 (93%) were seropositive (Figure 2B).

**Figure 3:**
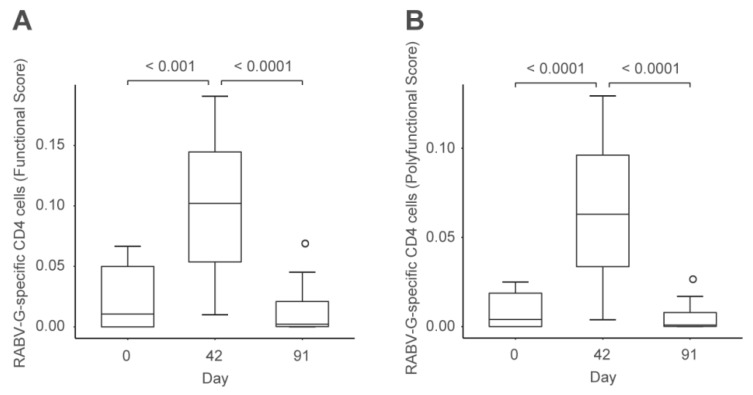
Cellular immune responses in human volunteers to intradermal injection of 80 μg mRNA (adapted from reference [34]). Intracellular cytokine staining of RABV-G-specific CD4+ T cells were analyzed using COMPASS and summarized as functionality (**A**) or polyfunctionality (**B**) scores. Boxes depict median values and IQRs, and whiskers extend from the hinge to the highest or lowest value within 1.5 × IQR of the respective hinge. *N* = 21 for days 0 and 42 and *n* = 15 for day 91.

Initial neutralizing responses correlated with IgM levels at 21 days postvaccination and with IgG levels at day 42, indicating the typical class-switching observed with inactivated vaccines. After the booster there was no change in IgM, and the response consisting of increased IgG, already apparent 7 days post vaccination, was consistent with a memory response. Peripheral blood mononuclear cells (PBMCs) were obtained at days 0, 42, and 91 from the same subjects shown in Figure 2 after receiving 80 μg by intradermal injector. These were used to assess multifunctional subject-level antigen-specific T-cell responses by intracellular cytokine staining, the data being analyzed as functionality scores using COMPASS software [36]. This showed a transient but significant increase in scores of RABV-G-specific CD4+ T cells at day 42, which then declined to baseline at day 91 (Figure 3), consistent with the contraction and memory phase of an immune response. There was a correlation between the humoral and cellular aspects of the immune response, confirming the first demonstration that RABV-G mRNA induced virus-specific cellular immune responses in humans.

This demonstration of immune responses in humans is not without limitations. Responses were clearly more reliant on the mode of administration rather than the route. Intradermal or intramuscular injection using standard needle and syringes to administer mRNA by either route did not result in immune responses. However, using needle-free injector devices for either of these routes did provide robust immune responses, with evidence of induction of both humoral and cellular immunity and immune memory. Reasons for this increased immune response with the jet injection device were not further investigated in this study. Similar observations have already been published demonstrating that the use of such an injector device leads to significantly higher levels of neutralizing antibodies in comparison to conventional i.d. or i.m. injection in the context of DNA vaccines in animal studies [37,38]. Others attributed this higher immune response using a needle-free injector to an increase in protein secretion and, therefore, to increase priming of APCs and T cell stimulation [39]. While this is a convincing demonstration of the concept of an mRNA rabies vaccine, the extent of this immune response and the reliance on specialized injector devices is not considered to be practical for routine prophylactic use. Further preclinical research was undertaken into the formulation of the mRNA to better understand the mechanism of action to be able to introduce improvements.

## 3. Formulation of Lipid Nanoparticle (LNP) mRNA

**Figure 4:**
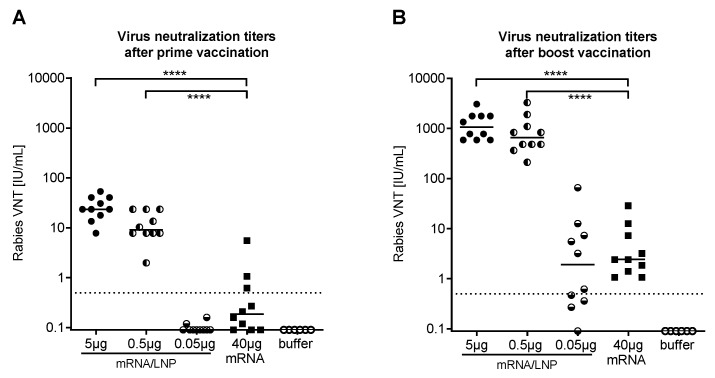
Serum neutralizing antibody responses to mRNA-LNP formulations in mice (adapted from reference [41]). (**A**) shows rabies virus neutralizing antibody titers (VNT) measured in serum of Balb/c mice three weeks after the second of two intradermal vaccinations given 3 weeks apart; vaccinations consisted of 0.05, 0.5, or 5 μg mRNA-LNP or 40 μg nonformulated mRNA or buffer. (**B**) shows VNT titers measured in serum of Balb/c mice two weeks after the mice received a booster dose of the same vaccine five months after the primary doses. Groups of ten mice were in each case. Dashed line shows WHO protective value (0.5 IU/mL), **** = *p* < 0.0001; Lipid nanoparticle (LNP)-formulated mRNA was generated using LNPs provided by Acuitas Therapeutics (Canada).

A key observation made by Geall et al. [40] was that replicating nonmodified mRNA molecules formulated as lipid nanoparticles (LNPs) were able to induce protective immune responses in mice against respiratory syncytial virus (RSV). The impact of LNP encapsulation of the rabies RABV-G nonreplicating mRNA proven in the studies described above was investigated by Lutz et al. [41]. Initially they investigated the effect of LNP on the activation of the innate immune system by measuring cytokine and chemokine concentrations following intramuscular injection into mice. There was no effect with buffer or plain mRNA, but the LNP mRNA formulation induced a marked, transient local increase in the proinflammatory factors, tumor necrosis factor (TNF) and IL-6 [41]. These chemokines have a known stimulatory effect on the recruitment and activation of immune cells creating an ideal environment for T and B cells to elicit immune responses against the associated antigen [42]. This was then investigated by measuring the VNT response in BALB/c mice following intramuscular administration of mRNA-LNP compared with plain mRNA or vaccine buffer (Figure 4). A 40 μg dose of plain RABV-G mRNA failed to elicit a VNT over the WHO threshold in the majority of the mice, whereas low doses, 0.5 or 5 μg, of LNP mRNA elicited over 50-fold increases in VNT three weeks after vaccination, when all mice had titers above the WHO predefined titer (Figure 4A). A second LNP mRNA vaccination three weeks later increased VNTs by two orders of magnitude to levels 250-fold higher than those observed in the plain 40 μg mRNA group (Figure 4B). These humoral immune responses were accompanied by marked increases in both CD4+ and CD8+ cells showing a cellular component to the immune response [41].

**Figure 5:**
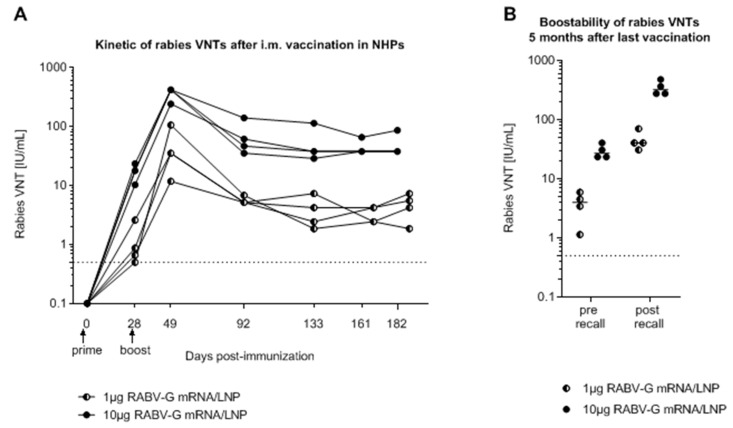
Kinetics of the serum neutralizing antibody responses to mRNA LNP formulations in nonhuman primates (NHPs) (adapted from reference [41]). (**A**) Rabies virus neutralizing antibody titers (VNTs) in individual cynomolgus monkeys (NHP) after two intramuscular doses of 1 or 10 μg mRNA LNP given 28 days apart. (**B**) Five months after the primary doses, VNTs were measured again before and after receiving a booster dose of the respective vaccine. The dashed line shows WHO protective value (0.5 IU/mL). Lipid nanoparticle (LNP)-formulated mRNA was generated using LNPs provided by Acuitas Therapeutics (Canada).

The kinetics of the response to LNP mRNA were investigated in nonhuman primates (NHPs, cynomolgus monkeys). A 1 μg dose of LNP mRNA was sufficient to induce titers above the 0.5 IU/mL threshold in most animals; a more pronounced response to a 10 μg dose resulted in VNTs above the WHO threshold in all animals after the first vaccination (Figure 5A). After a second dose administered 28 days after the first, there were further dose-dependent increases in VNTs that persisted above the WHO threshold for the six months of monitoring. A third “recall” vaccination administered at six months produced an anamnestic response to the peak levels observed after the second priming dose with rapid kinetics indicative of an established memory response to the encoded antigen (Figure 5B). Additional investigations showed that these humoral responses in NHPs to LNP mRNA were also accompanied by increases in rabies-specific T cell responses. Furthermore, these humoral and cellular immune responses were higher and stronger than those observed in NHPs following full human doses of licensed rabies vaccine (Rabipur) [41].

While difficult to assess in NHPs, the vaccinations seemed to be well tolerated, with no discernible pain at the injection site or variation in body temperature. Measurable indicators of reactivity including laboratory assessments (cytokine and chemokine concentrations), body temperature, and body weight showed only minor and transient variations in a similar range as responses to licensed vaccines [42]. These encouraging results with LNP mRNA are now being followed up by investigation in human trials; a clinical trial (ClinicalTrials.gov: NCT 03713086) started recruiting to assess one or two intramuscular injections of a new formulation of mRNA LNP, CV7202, in adults aged 18–40 years.

## 4. Hypothesized Mode of Action of LNP mRNA

**Figure 6:**
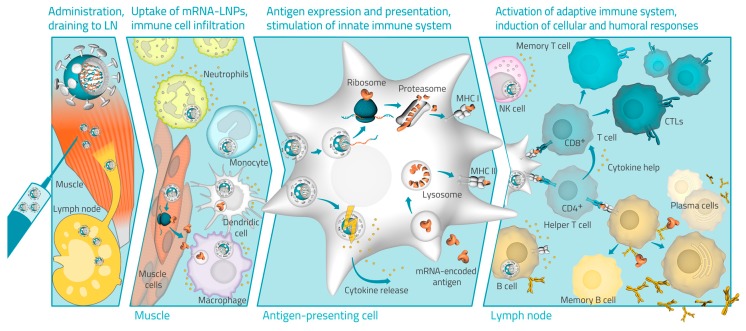
Schematic illustration of the mode of action of LNP mRNA formulations.

Data from preclinical studies in mice and NHPs have allowed some assessment of the putative mode of action of the LNP mRNA formulation, as depicted in Figure 6. Formulation in LNP encapsulates the naked mRNA in particles, which protect it against RNases when injected, facilitating and enhancing its uptake by endocytosis into cells surrounding the injection site [43]. Once released in the cytoplasm, the mRNA is expressed into the target RABV-G antigen inside transiently transfected cells, such as resident professional antigen-presenting cells (APCs), and induced neutrophils and nonleukocytic cells [23]. The intramuscular injection of the LNP-formulated mRNA vaccine also activates the innate immune system with transient local increases in proinflammatory cytokines, mainly tumor necrosis factor (TNF) and IL-6, producing an immunostimulatory environment at the injection site and in draining lymph nodes (dLNs) as demonstrated in mice [41]. The expressed antigenic peptides are then presented on MHC class I and MHC class II molecules of immunologically relevant cells. Expansion of the immune response is likely achieved by antigen expressed on the surface of APCs being recognized by CD4+ and CD8+ cells [44], which is supported by the measured cytokine response and increased levels of leukocytes in the dLN as demonstrated in mice [41]. The induction of high levels of antibody-producing B cells produces the humoral responses, which were measured as circulating RABV-G antibodies in the blood of vaccinated animals. The production of memory B and T cells leading to the booster responses against RABV-G that were observed in animals will also be investigated in humans.

## 5. Conclusions

Recent publications have described a technology platform for the flexible and accurate production of mRNA encoding for protein antigens that has been applied to rabies. Preclinical studies have demonstrated that intramuscular or intradermal injection of mRNA can induce humoral and cellular immune responses against the encoded antigen. However, in human volunteers, while the protamine-formulated mRNA had an acceptable reactogenicity profile, the immune response is dependent upon the technique used for administration. Preclinical studies in mice and cynomolgus monkeys show that a new vaccine candidate using lipid nanoparticle encapsulation of the mRNA induces both humoral and cellular immune responses that are significantly improved compared with the protamine formulated mRNA. Further studies are currently underway assessing LNP mRNA formulations in human volunteers to evaluate how results from promising preclinical studies can be used as a basis for the development of a novel prophylactic rabies vaccine for the human population.
